# Astigmatism and Its Consequences

**Published:** 1904-04-09

**Authors:** 


### Astigmatism and its Consequences.

The evil effects of astigmatism have recently been proclaimed to the profession in somewhat dramatic fashion, and an attempt has been made to establish the doctrine that to refractive errors of this order may be referred many, if not most, of the ills to which flesh is heir. It is not only clinical records, the result of direct observation, that have been advanced in support of this thesis. In addition, the biographies of many of the great men of the last century have been brought under scrutiny and have been analysed and reconstructed with a view to show that the personal sufferings and limitations of these men are to be traced solely and entirely to uncorrected astigmatic defects. It is impossible to refuse a tribute of admiration to the ingenuity and enthusiasm which have marked this procedure, and the good faith of those who are responsible for it is entirely beyond question. Yet it may reasonably be doubted whether the method is a sound one, and so far from producing disciples it has rather given occasion to the enemy to blaspheme. There have been so many similar simple explanations offered, each in its day, as the single cause of human suffering that the suggestion is suspect from the outset, and to those chastened by previous experience, astigmatism is thus apt to take rank with ulceration of the cervix, displacement of the uterus, enlargement of the prostate, and the presence of uric acid, as one more illustration of the errors produced by narrowness of outlook and ill-regulated enthusiasm. In the very nature of things there can be no proof that certain persons no longer under observation were the subjects of particular ocular defects. It is by actual experiences of to-day and not by conjectures regarding past events that the results of astigmatism and the benefits produced by the use of appropriate glasses must be estimated. In this regard attention may be directed to a recent annual report of one of the Colonies for Epileptics established in the United States. It appears that some 12 months ago the 68 members of this colony were subjected to an expert examination by physicians convinced of the relationship between epilepsy and ocular defects, and no less than 67 were found to be the subjects of "astigmatic and anisometropic defects." Accordingly in all these cases suitable glasses were prescribed. The procedure has now enjoyed a year's trial, and the physician in charge of the colony reports that "only one patient out of the 68 experienced any benefit in his disease while wearing glasses," and even in this case the mental condition of the patient was unimproved. Further, in two instances a distinct increase in the frequency of the epileptic seizures followed the use of the glasses prescribed. Of course it may reasonably be objected that the significance of this experience may be exaggerated. It is one thing to apply a therapeutic method to a series of cases of confirmed epilepsy and quite another and different thing to use the same method in those in whom this disease has only betrayed its early beginnings. But making this allowance in full, the experience indicates the danger which attends extravagant and exaggerated claims. For when these are shown by experience to fail in part, many will draw the inference that they have not even a measure of truth. To commend a proposition to men who have the scientific habit of mind it must be presented in a form which does not outrage probability, and must be supported by a series of exact observations which bear the mark of an impartial judgment. The cruel teachings of experience have repeatedly demonstrated how vast is the gap between the actual value of various forms of treatment and the value claimed for these by their sponsors. That in certain instances astigmatism is responsible for considerable personal discomfort, and even for more serious consequences, will be generally allowed. That in conditions where peripheral irritation may be the sole or a contributory cause the possible existence of astigmatism ought to be remembered, and the defect, if present, corrected, is a sound rule of practice. But the general adoption of this rule will be hindered rather than promoted by large propositions in which refractive errors are exalted to a commanding position among the causes of disease. In spite of the word of a great authority, we venture to doubt the accuracy of the opinion that astigmatism of less than a dioptre is seldom disturbing to vision, at least if by "vision" is meant ease and comfort in the use of the eyes. But we are even more convinced that sound doctrine and practice in this matter are far removed from the extravagance of some recent claims. These, by their very excess, are likely to alienate the sympathies of moderate and reasonable men.

